# NIR Photosensitizer for Two-Photon Fluorescent Imaging and Photodynamic Therapy of Tumor

**DOI:** 10.3389/fchem.2021.629062

**Published:** 2021-02-23

**Authors:** Lujia Chen, Meijuan Chen, Yuping Zhou, Changsheng Ye, Ruiyuan Liu

**Affiliations:** ^1^ Breast Center, Department of General Surgery, Nanfang Hospital, Southern Medical University, Guangzhou, China; ^2^ State Key Laboratory of Organ Failure Research, Guangdong Provincial Key Laboratory of Viral Hepatitis Research, Department of Hepatology Unit and Infectious Diseases, Nanfang Hospital, Southern Medical University, Guangzhou, China; ^3^ Guangdong Provincial Key Laboratory of Medical Image Processing, School of Biomedical Engineering, Southern Medical University, Guangzhou, China

**Keywords:** NIR emission, NIR photosensiziter, D-π-A structure, imaging-guided photodynamic therapy, two-photon fluorescent imaging

## Abstract

Preparation of near-infrared (NIR) emissive fluorophore for imaging-guided PDT (photodynamic therapy) has attracted enormous attention. Hence, NIR photosensitizers of two-photon (TP) fluorescent imaging and photodynamic therapy are highly desirable. In this contribution, a novel D-π-A structured NIR photosensitizer (TTRE) is synthesized. TTRE demonstrates near-infrared (NIR) emission, good biocompatibility, and superior photostability, which can act as TP fluorescent agent for clear visualization of cells and vascular in tissue with deep-tissue penetration. The PDT efficacy of TTRE as photosensitizer is exploited *in vitro* and *in vivo*. All these results confirm that TTRE would serve as potential platform for TP fluorescence imaging and imaging-guided photodynamic therapy.

## Introduction

Recently, photodynamic therapy (PDT) as a noninvasive treatment procedure has attracted enormous attention due to its selective destroy of local lesions (Dai et al., 2019; Li et al., 2016; Kwiatkowski et al., 2018). As an important element of PDT, photosensitizers transfer light energy to oxygen and generate reactive oxygen species (ROS), which destruct the morphology and function of cells, and ultimately result in cancer cell damage and apoptosis (Dai et al., 2020; Li et al., 2018; Zhou et al., 2016). Hence, the development of efficient photosensitizers has become the focus of attention, and various kinds of photosensitizers have been produced (Wang et al., 2018a; Xiao et al., 2020; Huo et al., 2020; Lindem and Vazquez, 2020).

As a noninvasive biological imaging modality, NIR fluorescence imaging techniques supplies powerful tool to visualize cell biological events from molecules levels, subtle cellular structures to complete organisms with high spatiotemporal resolution (Kim et al., 2017; Kobayashi et al., 2010; Li et al., 2018; Hu et al., 2020). However, fluorescent imaging has some limitations including high photodamage, low penetration, and high photobleaching. Compare to conventional fluorescence imaging technology, two-photon (TP) fluorescence imaging exhibits various merits such as low photodamage, deep penetration, high spatial resolution, and has attracted much attention for use in intravital imaging of vasculature and tissues (Kim and Cho, 2015; Kuo et al., 2020; Qin et al., 2020).

Hence, in terms of photosensitizers, the coupling of ROS production with NIR emission has been utilized for imaging-guided PDT, which has acted as a promising alternative for cancer treatment (Shen et al., 2011; Wang et al., 2017; Zhu et al., 2017; Dudek et al., 2020; Yan et al., 2021). An ideal photosensitizer for imaging-guided PDT should possess properties, such as negligible dark toxicity, bright NIR emission, good photostability, ROS generation capacity, and biocompatibility (Li et al., 2018; Sarcan et al., 2018). In recent years, various NIR photosensitizers have been prepared for imaging-guided PDT of tumor, including porphyrin, chlorin, phthalocyanine, and BODIPY derivatives (Liu et al., 2016; Pan et al., 2019; Szurko et al., 2020; Zheng et al., 2020a). However, these NIR photosensitizers suffer from several intrinsic drawbacks, such as small Stokes' shift, poor photostability, and unsatisfied biocompatibility. Thereby, it is meaningful to develop new NIR photosensitizers for photodynamic therapy of tumor.

NIR fluorophores containing D-π-A structure have been proven to be an excellent candidate for imaging-guided photodynamic therapy owing to the NIR emission and high ROS generation efficient (Leitl et al., 2014; Liu et al., 2018; Yuan et al., 2020). Besides, photosensitizers with D-π-A structure have strong intramolecular charge transfer (ICT), which reduce electronic bandgaps, extend absorption and emission wavelengths, enhance the two-photon absorption properties of fluorophore, and facilitate ROS generation (Pawlicki et al., 2009; Wu et al., 2017; Niu et al., 2019a; Niu et al., 2019b; Lu et al., 2020; Wan et al., 2020). In recent times, efforts have been made to increase intramolecular charge transfer effect of D-π-A structured photosensitizers (Deraka et al., 2017; Chai et al., 2019; Samanta et al., 2019). To this effect, various electron-deficient units have been widely explored, such as pyduium (Shi et al., 2020; Zheng et al., 2020b), benzo [c] (Li et al., 2016; Li et al., 2018; Dai et al., 2019), thiadiazole (Guo et al., 2017; Zhou et al., 2020), rhodamine (Tang et al., 2018; Lv et al., 2019), indaceno (Wang et al., 2016), and tricyanofuran (Wu et al., 2019). Among them, rhodanic, an electron-deficient core, can serve as block to build NIR fluorophore (Wan et al., 2017; Wang et al., 2018b; Xia et al., 2018). However, rhodanic molecules face some challenges, such as low absorption in the NIR region and limited ROS generation efficiency. Hence, it is highly desirable to design new photosensitizers containing rhodanic with high PDT performance.

In this contribution, we develop a D-π-A structured NIR photosensitizer (TTRE), which was rationally designed as electron-donating triphenylamine as electron-donating group, rhodanic as electron-withdrawing units, and thiophenyl as *π* bridge. TTRE exhibited NIR emission (around 680 nm), ROS generation ability, and two-photon fluorescent imaging capacity. Both *in vitro* and *in vivo* studies confirmed that TTRE has effective anticancer potential and is amenable to imaging-guided photodynamic therapy of tumor.

## Material and Methods

### Materials

All the solvents and reagents utilized in this contribution were of analytical grade. 5-(4-(Diphenylamino) phenyl) thiophene-2-carbaldehyde, 2-ethylhexyl 2-cyanoacetate, 4-isothiocyanatobenzonitrile, DBU, and ethyl bromoacetate were purchased from 3A Chemical Co. Ltd. The biological chemical reagents containing ROS indicators of 9,10-anthracenediyl-bis(methylene)-dimalonic acid (ABDA) and 2′,7′-dichlorodihydrofluorescein diacetate (DCFDA) were offered from aladdin Co., Ltd. DAPI and Annexin V-FITC apoptosis detection kit were purchased from Beyotime biotechnology Co., Ltd.

### Instruments

NMR spectra were measured via Bruker 400 MHz NMR with CDCl_3_ and DMSO-d_6_. UV absorption spectra were recorded on Thermofisher Evolution 300 spectropolarimeter. Fluorescent spectra were obtained using Thermofisher Lumina spectrofluorometer. Infrared (IR) spectroscopy was performed with Shimadzu FTIR-8100 spectrophotometer. High resolution mass spectra were obtained on Bruker Autoflex instrument. Confocal laser scanning microscope (CLSM) images were performed on Olympus FV1000-IX81 confocal laser scanning microscope. Two photon fluorescence imaging was obtained using upright multiphoton microscope (FVMPE-RS, Olympus, Japan). Small animals’ fluorescence imaging was carried out by Bruker FX Pro living imaging system.

### Synthesis of Rhodanic

DBU (3.04 g, 20 mmol), 2-Ethylhexyl 2-cyanoacetate (3.94g, 20 mmol), and 4-isothiocyanatobenzonitrile (3.52 g, 22 mmol) were added to CH_3_CN (50 ml) at room temperature. After stirred for 30 min, ethyl bromoacetate (5.65 g, 34 mmol) was added to the mixture. The mixture was refluxed for 8 h. The CH_3_CN was evaporated. The solid was acidified with 1 M HCl (60 ml) and extracted with dichloromethane. The organic layer was concentrated, then recrystallized in CH_3_CN to produce pale yellow solid (6.43 g, 81%). ^1^H NMR (500 MHz, DMSO-d_6_) *δ*(ppm) 8.05∼8.07(d,2H), 7.67∼7.69 (d, 2H), 4.24∼4.25(t, 2H), 4.00∼4.02(t, 2H), 1.28∼1.44(t, 2H), 1.21∼1.26(m, 2H), 1.28∼1.44(m, 1H), 1.21∼1.26(m, 8H), 0.81∼0.86(m, 6H). ^13^C NMR (100 MHz, DMSO-d_6_) *δ* (ppm) 173.60, 172.24, 165.22, 139.40, 133.86, 131.29, 118.56, 113.65, 112.72, 88.92, 76.47, 67.38, 38.57, 32.72, 30.06, 28.64, 23.56, 22.75, 14.25, 11.21.

### Synthesis of TTRE

5-(4-(Diphenylamino) phenyl) thiophene-2-carbaldehyde (1.77 g, 5 mmol), Rhodanic (1.985 g, 5 mmol), and CH_3_COONa (500 mg) were added to acetic acid (30 ml). The mixture was refluxed at 160 °C for 12 h. After cooling to room temperature, the solid was filtered and washed with cold MeOH. The solid was recrystallized from CH_2_Cl_2_/ethanol (1:10, v/v) to give TTRE as red solid. Yield: 2.97 g (81%). ^1^H NMR (500 MHz, CDCl_3_) *δ*(ppm) 8.05(s, 1H), 7.89∼7.91(d, 2H), 7.56∼7.58(d, 2H), 7.51∼7.53(d, 2H), 7.32∼7.35(m, 8H), 7.09∼7.19(m, 6H), 4.19∼4.22(m, 2H), 1.58(s, 1H), 1.28∼1.40(m, 8H), 0.86∼0.93(m, 6H). ^13^C NMR (100 MHz, CDCl_3_) *δ*(ppm) 178.00, 167.34, 163.97, 146.90, 142.47, 138.78, 136.86, 134.51, 133.62, 130.11, 129.45, 129.18, 128.72, 128.50, 127.43, 126.40, 125.84, 125.13, 123.90, 122.35, 117.81, 108.68, 103.12, 66.65, 39.10, 29.65, 29.27, 23.61, 22.84, 14.08, 10.94. IR(KBr) *v* (cm^-1^), 3422, 2963, 2925, 1719, 1578, 1527, 1491, 1437, 1367, 1325, 1293, 1154.

## Result and Discussion

### Synthesis and Properties of TTRE

**FIGURE 1:**
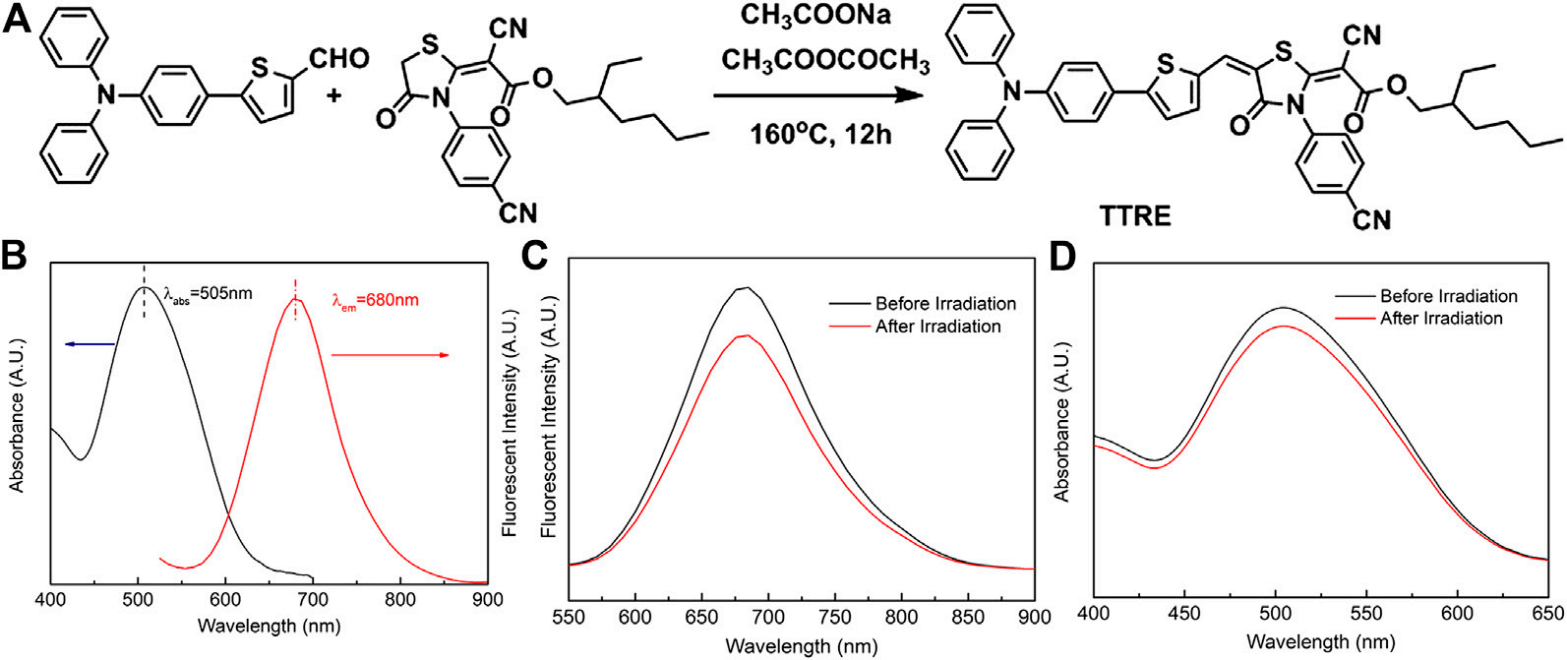
**(A)** The synthetic routine of TTRE. **(B)** The Normalized absorption and fluorescent spectra of TTRE in Water. **(C)** Fluorescent spectra of TTRE after irradiation (300mW/cm^2^). **(D)** UV spectra of TTRE after irradiation (300mW/cm^2^).

The D-π-A structure could reduce electronic band gaps and extend absorption/emission wavelengths of fluorophore. In addition, D-π-A structure fluorophore exhibit the two-photon absorption and ROS production. Herein, rhodanic and triphenylamine were attached to thiophenel group to build NIR photosensitizer TTRE (Figure 1A). The NMR, and IR spectra are listed in Supplementary Figure S1–S5 (Supporting Information).

TTRE’s optical properties was analyzed using UV-vis and fluorescent spectroscopy. As shown in Figure 1B, the absorption is centered around 505 nm in water containing 0.1% DMSO, while the emission maximum of TTRE is located in 680 nm which belongs to the near-infrared region. More importantly, TTRE emits NIR fluorescence with a large Stokes shift of 175 nm which enable it to give great advantage for bioimaging applications. Analysis of TTRE’s optical properties in various solvents using UV-vis and fluorescent spectroscopy was carried out. As shown in Supplementary Figure S6, the absorption maximum of TTRE varied from 475 to 525 nm in the different solvent. On the other hand, the emission maximum shifted from 600 to 680 nm. All these results confirmed that the optical properties of TTRE are strongly dependent on the solvent polarity. We also measured the fluorescent properties of TTRE in DMSO/toluene mixtures at various toluene concentrations (Supplementary Figure S7). TTRE exhibited weak emission in DMSO and fluorescence increased with gradual addition of toluene. Fluorescent intensity rose 12-fold at pure toluene relative to pure DMSO. These data show that TTRE is AIE active.

Photostability is critical for fluorescence imaging and photodynamic therapy. Here, the photostability of TTRE was examined (Figures 1C,D). After white light irradiation for approximate 10 min (300 mW/cm^2^), TTRE’s fluorescence reduced modestly, to 83% of the initial value, while its absorption spectrum still keeps 92% of original value, indicating TTRE has superior photostability.

### ROS Generation

To investigate the cytotoxicity of TTRE in dark or upon light irradiation, CCK-8 analysis was carried out. As shown in Supplementary Figure S8, the cytotoxicity of 4T1 cells is little in the absence of light. However, cell viability reduced to 15% after incubation with TTRE (10 µM) and white light irradiation (8 min, 60 mW/cm^2^), suggesting TTRE may be amenable to photo triggered therapy.

**FIGURE 2:**
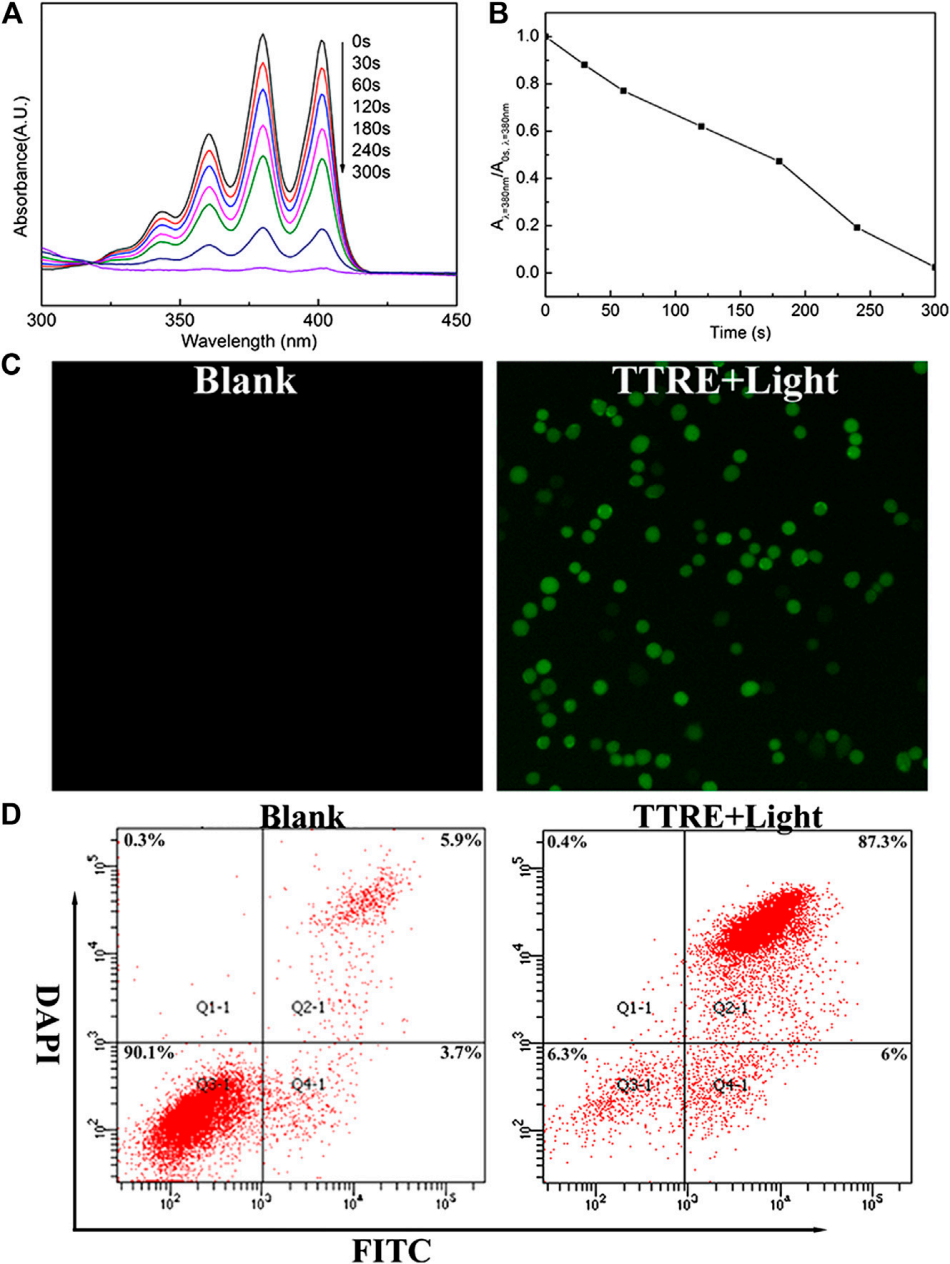
**(A)** UV-vis spectra change of ABDA and TTRE with different irradiation time of white light (60 mW/cm^2^). **(B)** Plots of *A*/*A*_0_ at 378 nm of ABDA *vs.* different irradiation times. *A*_0_ is the absorption of ABDA without irradiation, and *A* is the absorption with various irradiation time. **(C)** Intracellular ROS detection using DCFDA in 4T1 cells incubated with TTRE after white light irradiation. **(D)** Representative FCM profiles of 4T1 cells with different treatment.

TTRE’s capacity of ROS production was initially evaluated under white light irradiation (60 mW/cm^2^) with ABDA as ROS indicator **(** Figures 2A,B). Under light irradiation, the absorbance in 378 nm of ABDA solution rapidly fell in the presence of TTRE, suggesting highly efficient ROS production. To detect in cellular ROS generation, DCFDA was utilized as indictor (Figure 2C). Green emission was observed from the cells treated with DCFDA and TTRE, while no obvious fluorescence was detected in the absence of TTRE. It seems that TTRE efficiently products ROS in 4T1 cells. Double staining with Annexin V-fluorescein isothiocyanate (FITC) and DAPI was carried out to investigate the extents of apoptosis or necrosis after PDT with TTRE. The apoptosis ratio induced by TTRE and irradiation was up to ∼87.3%, which was significantly higher than in Blank group (Figure 2D). All these results confirm that TTRE could be a potential photosensitizer.

### NIR and Two-Photon Fluorescent Imaging

**FIGURE 3:**
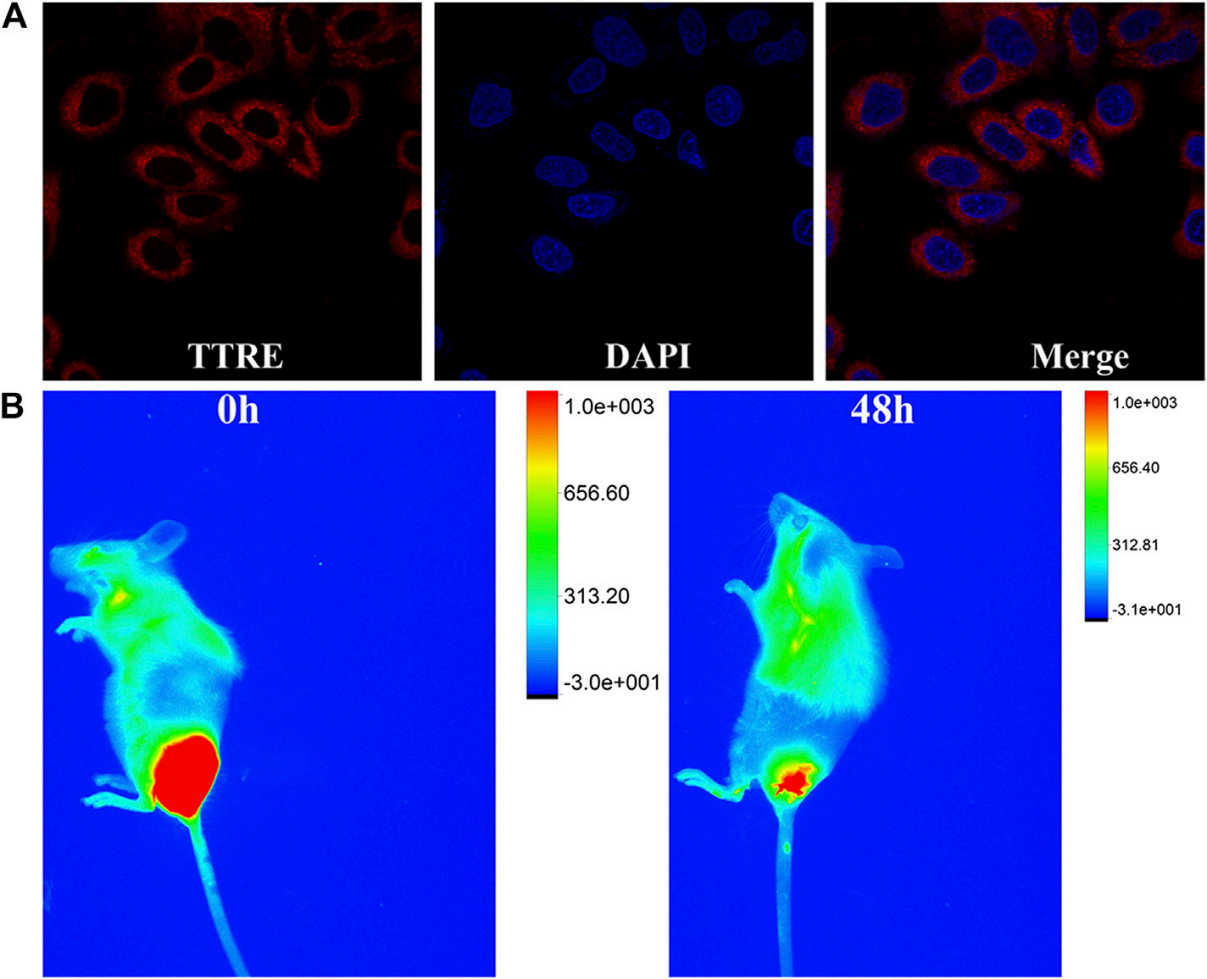
**(A)** Fluorescent imaging of 4T1 cells coculture with TTRE. **(B)** The fluorescent imaging of 4T1 tumor-bearing mice after intratumorally injection of TTRE *in vivo*.

NIR fluorescent imaging behaviors of TTRE in living cells was first investigated. As described in Figure 3A, NIR fluorescence within 4T1 cells can be detected, confirming the endocytosis of TTRE in 4T1 cells. To confirm the lysosomal specificity of TTRE, the colocalization experiment was carried out by incubating 4T1 cells with TTRE and Lyso-Tracker Green, which is commercial probe for lysosomal imaging. The red fluorescence of TTRE was overlapped with the green fluorescence of Lyso-Tracker Green. These data confirmed that TTRE permeates the cell membrane and accumulates in the lysosome.

Moreover, *in vivo* fluorescent imaging on tumor-bearing mice was carried out. As shown in Figure 3B, bright NIR fluorescent was detected at the tumor site after intratumorally injection of TTRE. Interestingly, NIR signal could be still examined after 48 h, confirming extended tumor retention. These data suggest that TTRE was suitable for fluorescent imaging-guided photodynamic therapy.

**FIGURE 4:**
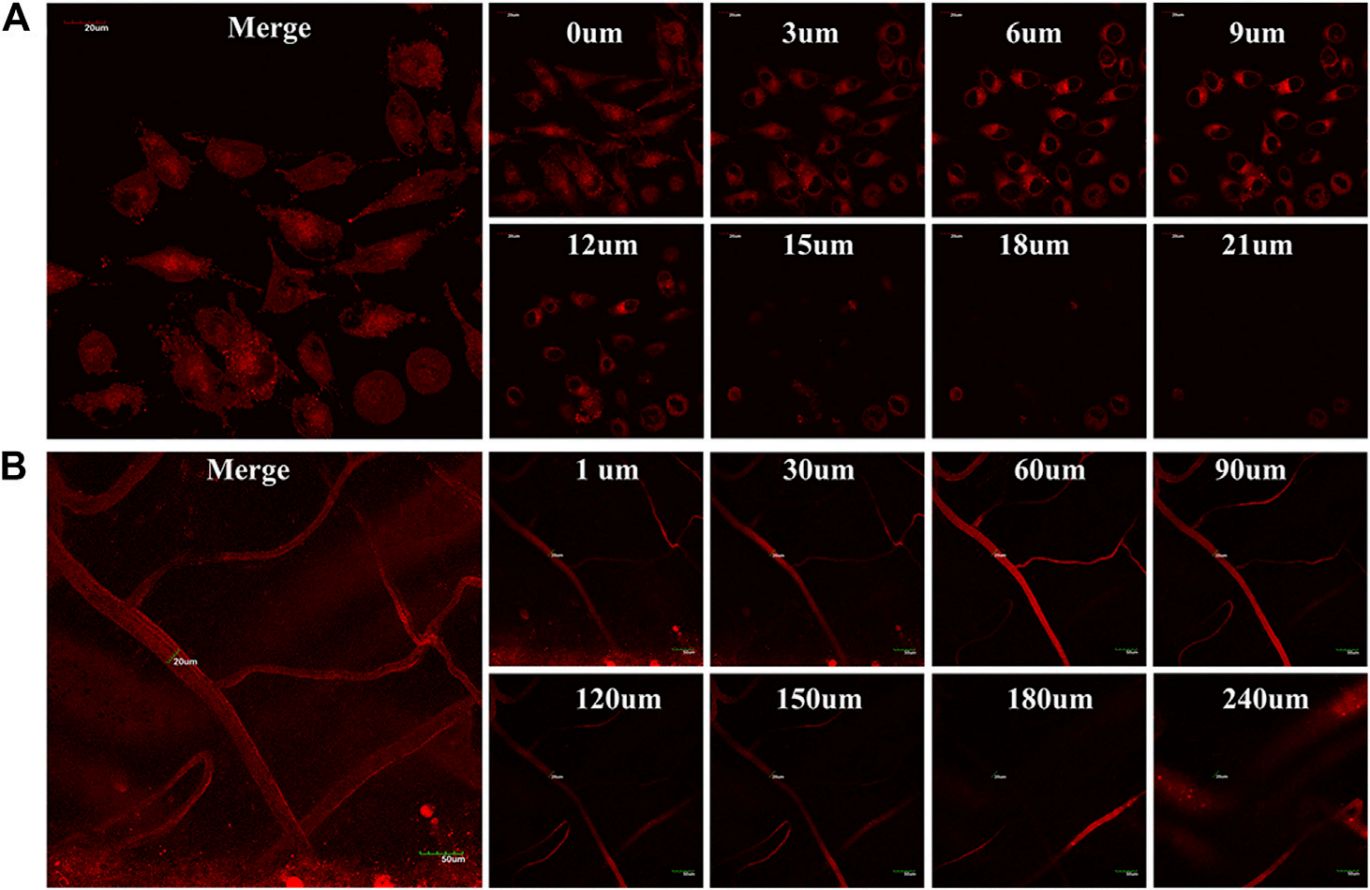
**(A)** TP fluorescent imaging of TTRE in living cells **(B)** TP fluorescent imaging of vascular in liver.

Given the TTRE enhances deep penetration and high contrast imaging, the performance of TTRE was measured using TP fluorescent imaging *in vitro*. Results shown in Figure 4A reveal the two-photon fluorescent imaging of TTRE even penetration 21 μm in cells. Therefore, TTRE was utilized to achieve deeper blood vascular imaging in mouse liver. Figure 4B show representative vascular images of the mouse liver at penetration depths from 1 to 240 μm. The fluorescent signal of TTRE can be detected at depths of up to 240 μm. The high-resolution 3D image *in vivo* provided clear spatial map of the major vascular networks and the details of tiny capillaries. All these results demonstrated that TTRE is promising two-photon fluorescent imaging platform.

### Inhibition of Subcutaneous 4T1 Tumors

**FIGURE 5:**
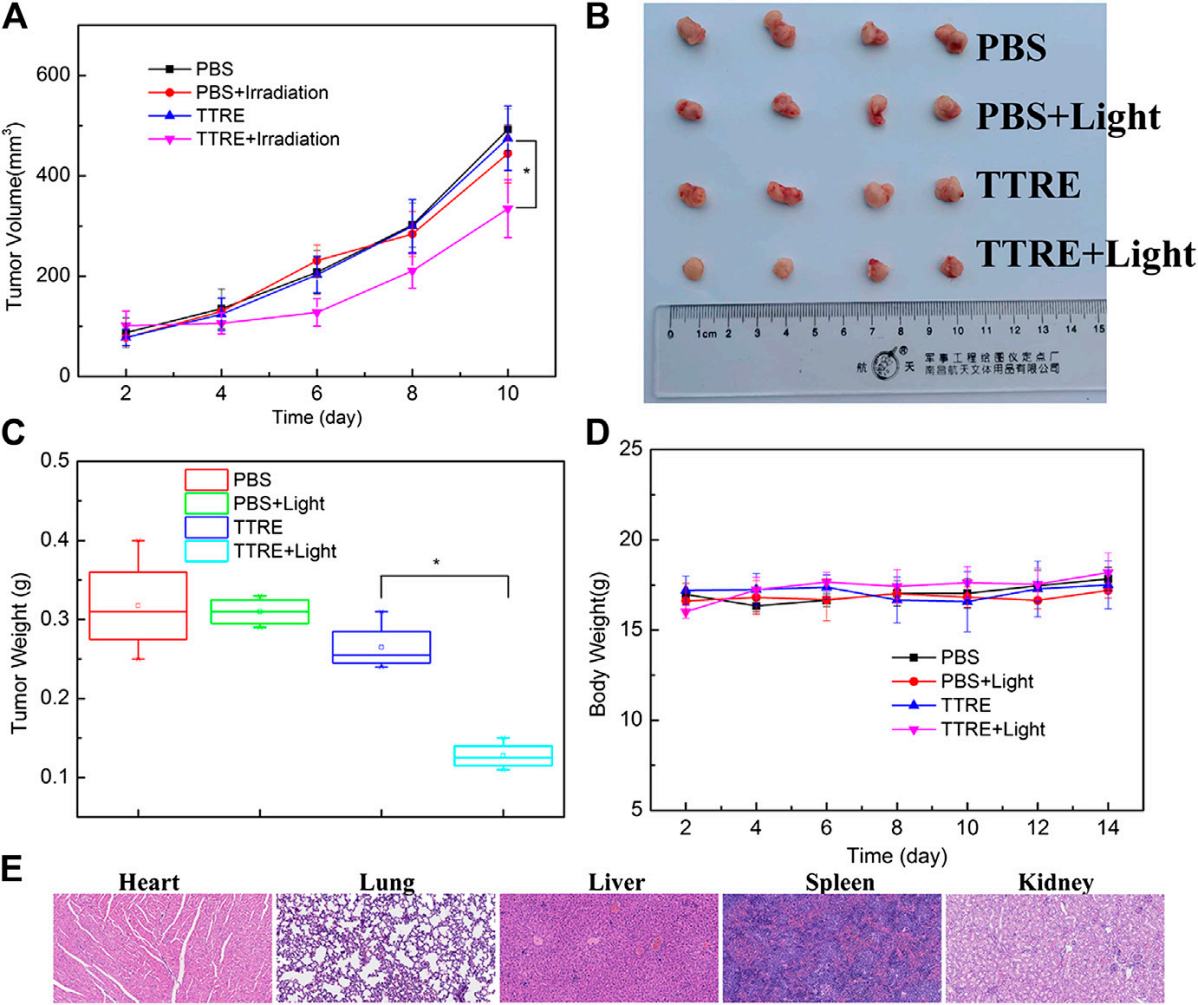
**(A)** Tumor volumes under different treatments; **(B)** Photos of typical tumor after different treatments; **(C)** Tumor weight under different treatments; **(D)** Body weights of the mice under different treatments; **(E)** H&E stained sections of heart, lung, liver, Spleen and Kidney.

For the investigation of the PDT property of TTRE *in vivo*, the 4T1 tumor-bearing mouse models were constructed, which were randomly divided into four groups and given different treatments (PBS, PBS with light, TTRE and TTRE with light). After being subjected to different treatments, the tumor volumes and tumor weights were monitored. A shown in Figures 5A–C, slight tumor growth inhibition was observed in the groups of PBS, PBS with light and TTRE, while TTRE with light group exhibited inhibitory effect on tumors, indicating that TTRE has good therapeutic effect under light irradiation. Importantly, during the treatment, all mice showed no significant abnormal changes in body weight (Figure 5D), and no significant damage in all major organs including the heart, liver, spleen, lung, kidney, and tumor (Figure 5E), thereby confirming the high biocompatibility and safety of TTRE for biomedical applications.

## Conclusion

In summary, a D-π-A structured NIR photosensitizer, TTRE, has been developed to realize photodynamic therapy. TTRE exhibited good biocompatibility, high photostability, and NIR emission property. TTRE was utilized as an efficient and effective photosensitizer for imaging-guided PDT with TP fluorescent imaging property. The excellent PDT performance of TTRE was further examined *in vivo*. This work provides insight into developing NIR photosensitizer for imaging-guided photodynamic therapy of cancer.

## Data Availability

The original contributions presented in the study are included in the article/Supplementary Material, further inquiries can be directed to the corresponding authors.
