# Supplementary material for: NIR Photosensitizer for Two-Photon Fluorescent Imaging and Photodynamic Therapy of Tumor
Source: Front Chem. 2021 Feb 23;9:629062. doi: 10.3389/fchem.2021.629062 (PMC7940671; doi:10.3389/fchem.2021.629062)
Supplement: Supplementary file 1 [file table1.doc]

Supporting Information

**NIR photosensitizer for two-photon fluorescent imaging and photodynamic therapy of tumor**

*L. Chen, M. Chen, Y. Zhou, C. Ye, R. Liu*

**Cellular endocytosis and Cytotoxicity**

4T1 cells were seeded in confocal cell dishes and cultured overnight to a confluence of around 80%. TTRE were added into the cell dishes. After incubation for 2 h, cells were washed with 1× PBS and taken for confocal imaging. The fluorescence was excited by 500 nm laser and collected within 600~700 nm.

For the cytotoxicity study, 4T1 cells were seeded in 96-well plates (5 × 10^3^ cells per well) and cultured overnight. Fresh culture media containing various concentrations of TTRE were added into the cell wells and incubated for 4 h. Afterward, fresh culture medium was added into the cell dishes after washing with 1× PBS for three times. Subsequently, the cells were subjected to white light irradiation (400~700 nm, 60 mW/cm^2^, 8 min) and incubated for an additional time of 20 h. Finally, the relative cell viabilities were evaluated by CCK-8 assay based on the manufacture’s instruction.

**ROS generation detection**

ABDA stock solution was added to TTRE solution (2μM) and white light (400~700 nm, 60 mW/cm^2^) was employed as the irradiation source. The absorption of ABDA at 375 nm was recorded at various irradiation time to obtain the decay rate of the photosensitizing process.

**Intracellular ROS Investigation**

4T1 cells were seeded in confocal cell dishes and cultured overnight to a confluence of around 80%. TTRE (2μM) were added into the cell dishes. After incubation for 4 h, cells were washed with 1× PBS and incubated with DCFDA (20 mM) for 30 min. The cells were then washed with 1× PBS and subjected to white light irradiation for 2 min (400~700 nm, 60 mW/cm^2^). Finally, the cells were taken for confocal fluorescence imaging. The excitation wavelengths were 480 nm for DCFDA. Fluorescence emissions are collected with 510~530 nm for DCFDA.

**Live/Dead Assay and Flow Cytometry Analysis of Light-Induced Cell Apoptosis.**

4T1 cells were seeded in six well dishes in 2 mL of DMEM cell culture media with a density of 1 ×10^6^ cells per well, and TTRE (2μM) was allowed. After laser irradiation as in the above-mentioned method, the cells were incubated for another 12 h and stained with Annexin V−FITC/DAPI apoptosis detection kit for further flow cytometry analysis.

Fluorescence signals in FITC channel and DAPI channel were measured for 5000 cells on a flow cytometer of Image StreamX Mark II (Merck Millipore, Seattle, WA).

**Two-photon Fluorescence Imaging in Cells and Tissues.**

The *in vivo and in vitro* two-photon fluorescence imaging experiments were performed on an upright multiphoton microscope (FVMPE-RS, Olympus, Japan) equipped with a water immersion objective (25×, NA: 1.05, XLPLN25XWMP2) specially designed for multiphoton excitation. Excitation wavelength = 1100 nm; emission filter = 600~700 nm. 4T1 cells used for two-photon microscopy were stained with TTRE (2 μM) refer to the procedure described for confocal fluorescence imaging.

Balb/c mice (female, 10~12 weeks old, around 25 g) were used for *in vivo* two-photon fluorescence imaging of liver. TTRE (100 μL, 2g/L) was intravenous injected into Balb/c mice. Mice were euthanized, and the liver samples were collected at 3 h after injection. Two-photon Fluorescence Imaging were collected.

**Animals and tumor models**

All animal experiments were approved by Animal Ethics Committee of Southern Medical University. Female Balb/c mice (4 ~5 weeks) were supplied by Guangdong Medical laboratory Animal Center. 1 × 10^7^ 4T1 cells were injected subcutaneously into the selected positions to establish the tumor model of Balb/c mice. Tumors were allowed to grow to about 80 mm^3^ in volume before used for *in vivo* imaging and photodynamic therapy.

***In vivo* PDT**

For *in vivo* evaluation of the PDT module, the tumor-bearing mice were randomly divided into four groups: (1) PBS; (2) light irradiation; (3) TTRE; (4) TTRE with light irradiation. Each mouse was intratumorally injected with 20 μL of TTRE (2 g/L) or 20 μL of PBS. 2 h After every injection, the tumor site was irradiated (100 mW/cm^2^, 8 min). Tumor volumes and body weights of all mice were monitored every 2 days. Tumor volumes of all mice were measured using a vernier caliper; the maximum diameter (length) and the largest transverse diameter (width) were used to calculate the tumor volume, V = width^2^ × length/2.

**Histological Examination**

Balb/c mice were intravenously injected with 100 μL of TTRE (0.5 mg/mL in 1× PBS), and major organs including kidney, lung, spleen, liver, and heart were collected and stained with hematoxylin and eosin (H&E) after two days administration. The images were taken by an upright microscope (Olympus BX43) with a 100× oil objective (UPlanSApo, NA: 1.40).


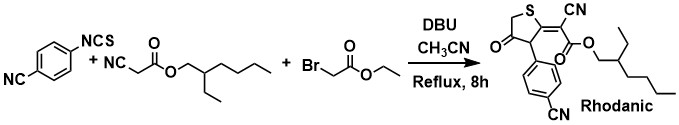


Scheme S1 The Synthesis routine of Rhodanic


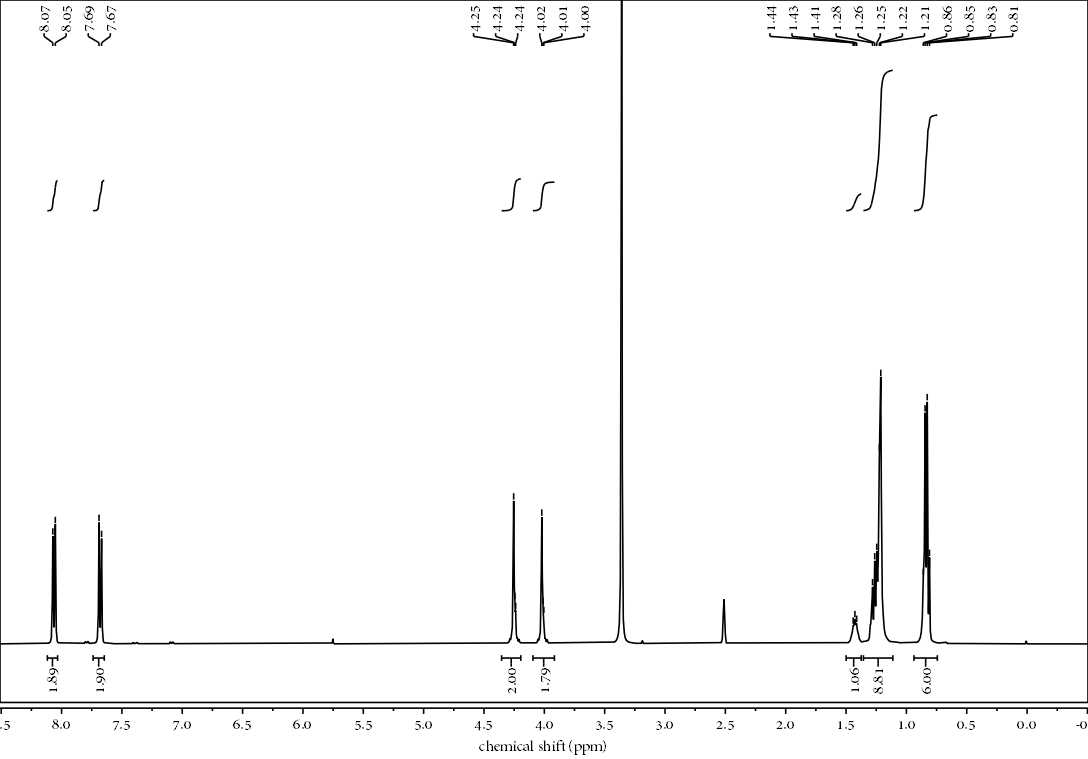


Figure S1 The ^1^H NMR Spectrum of Rhodanic


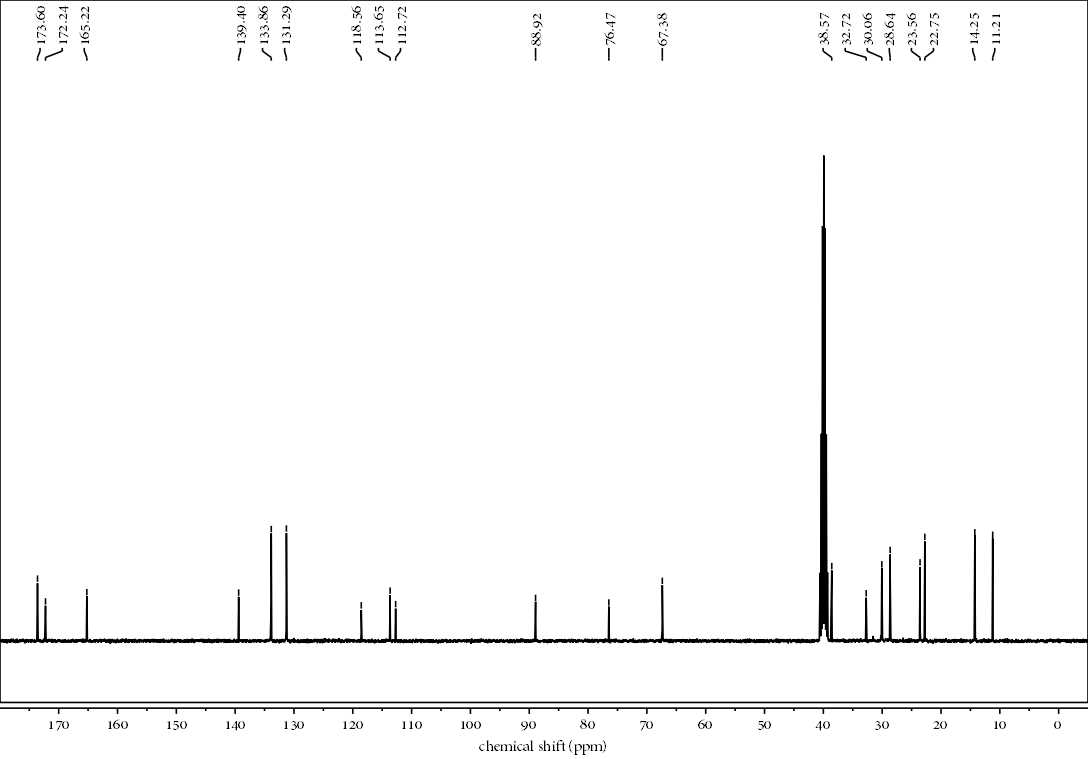


Figure S2 The ^13^C NMR Spectrum of Rhodanic


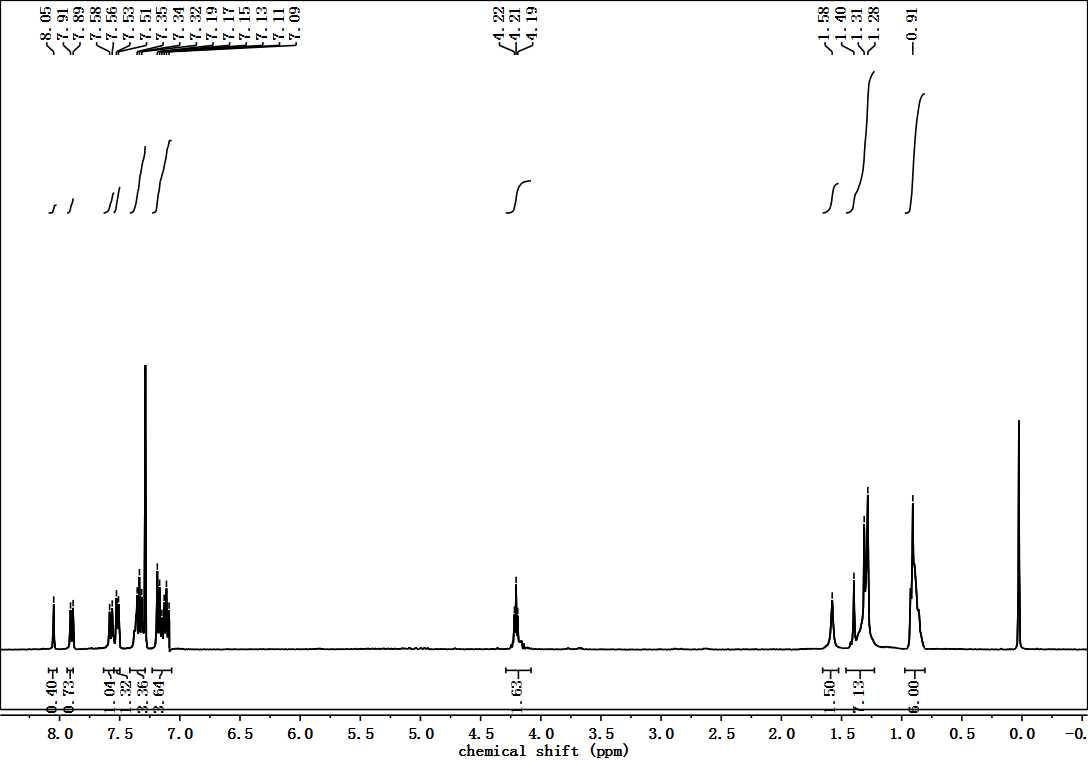


Figure S3 The ^1^H NMR Spectrum of TTRE


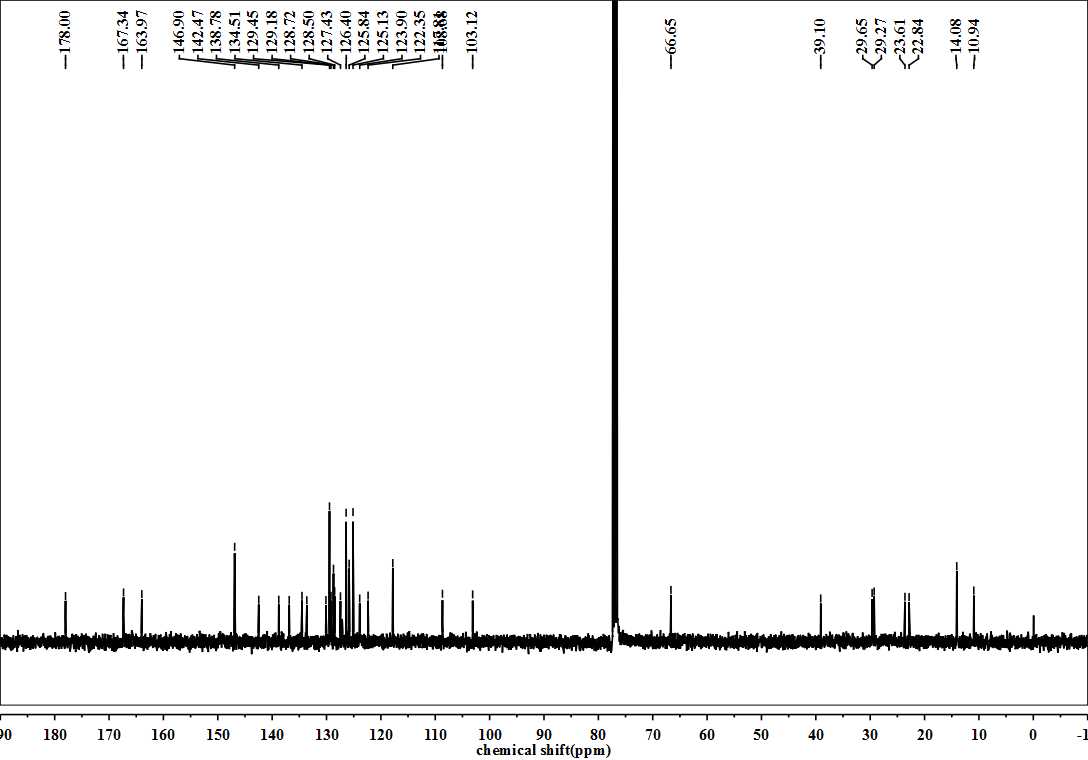


Figure S4 The ^13^C NMR Spectrum of TTRE


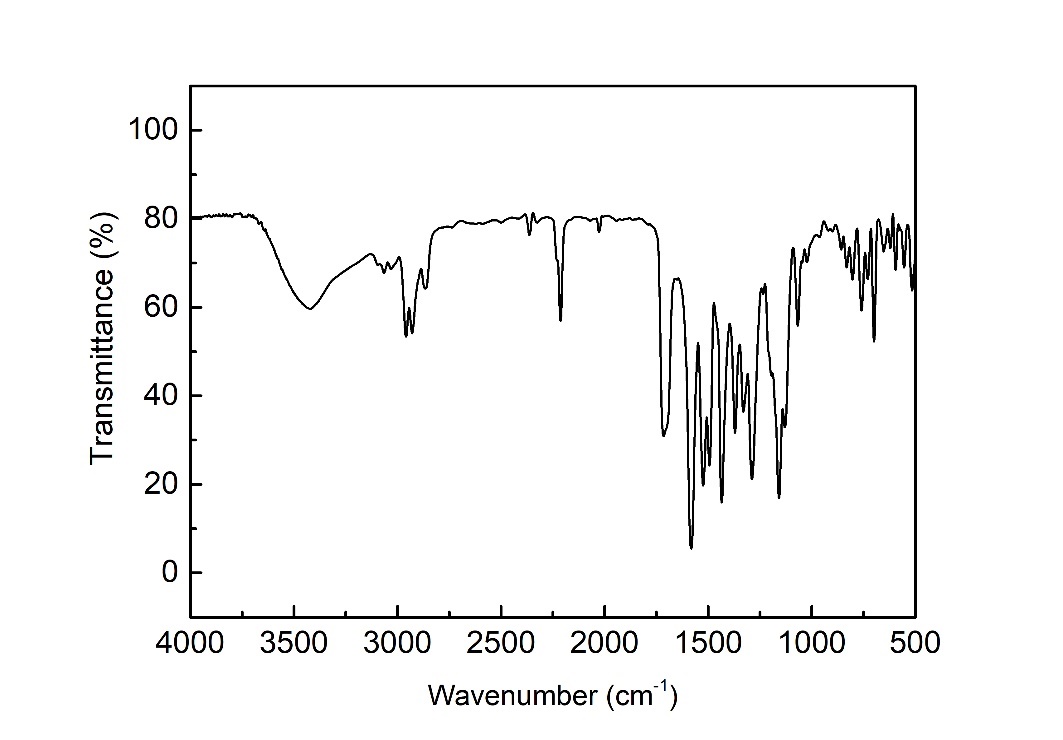


Figure S5 The IR Spectrum of TTRE


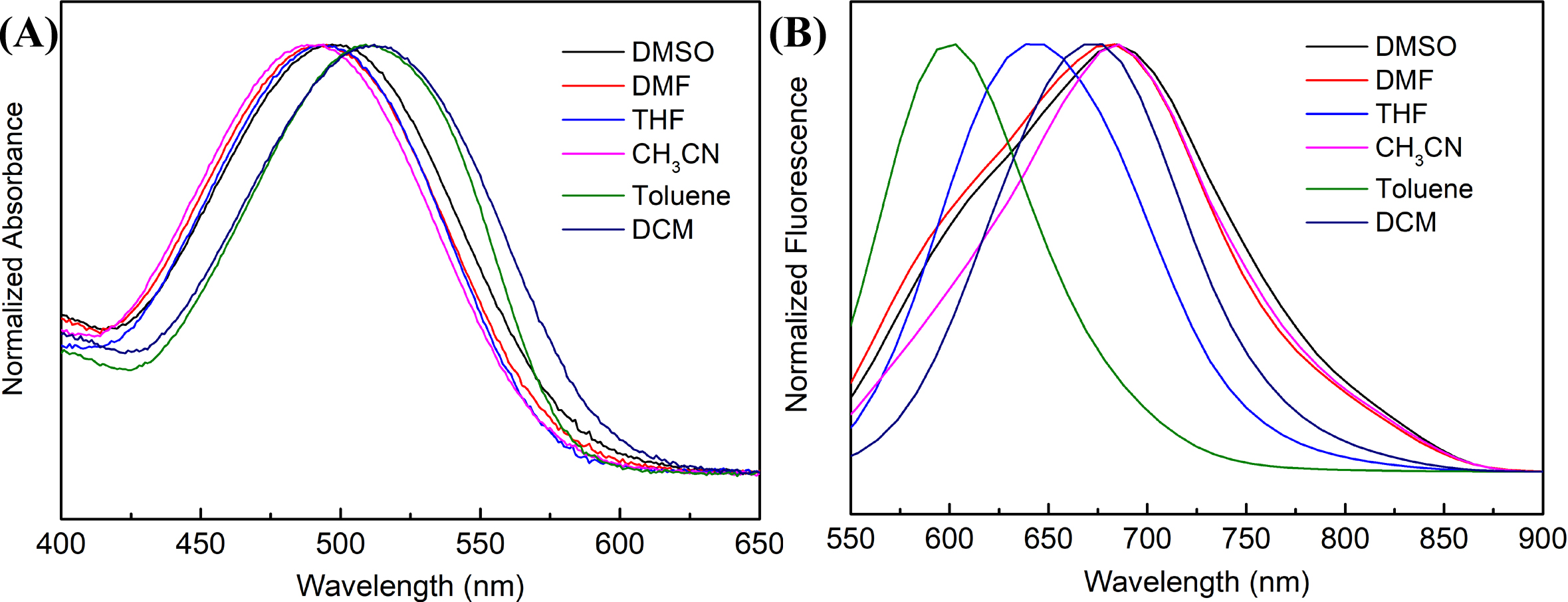


Figure S6 The Fluorescent and Absorbant Spectrum of TTRE in different solvent


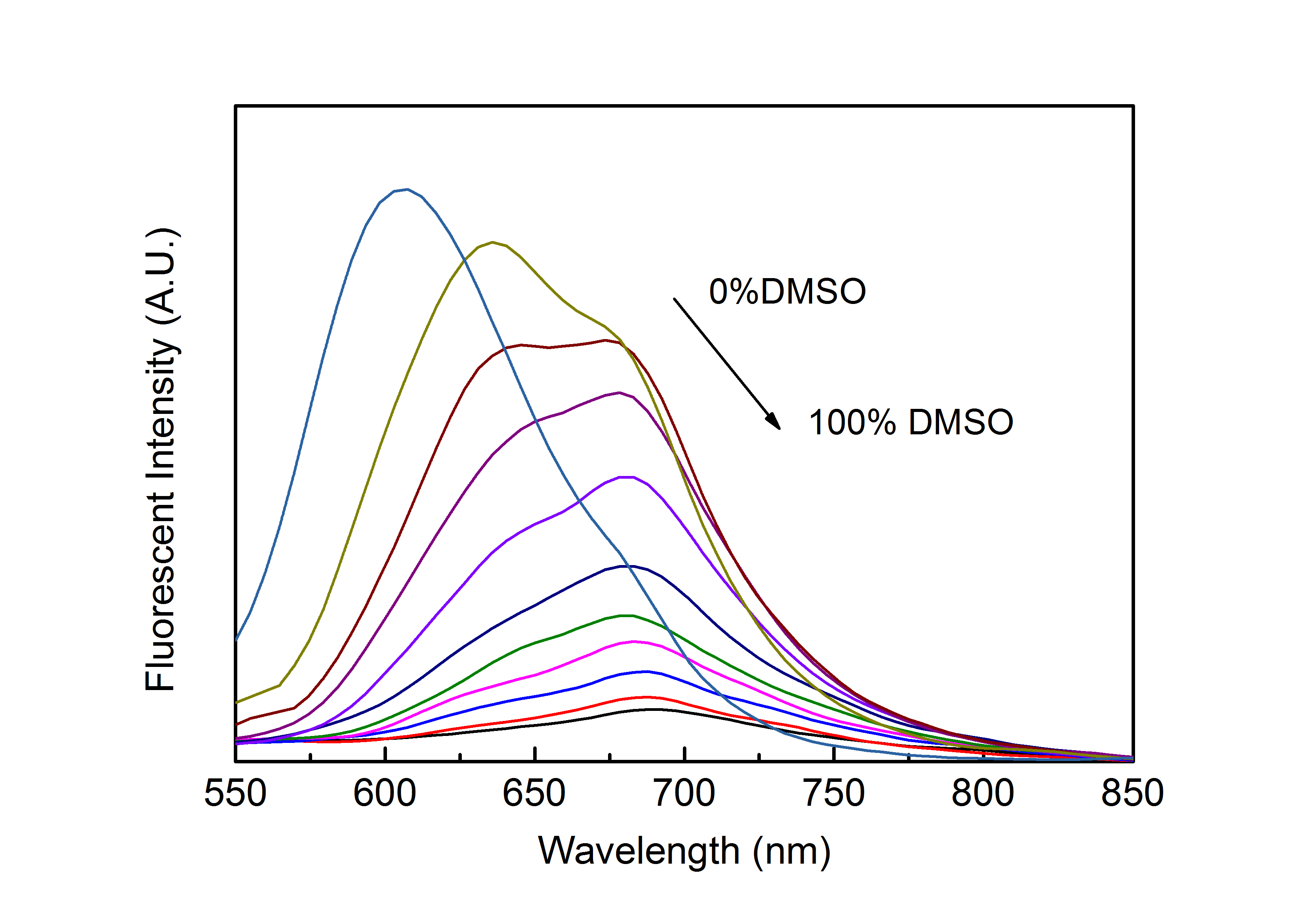


Figure S7 The fluorescent properties of TTRE in DMSO/toluene mixtures at various toluene concentrations.


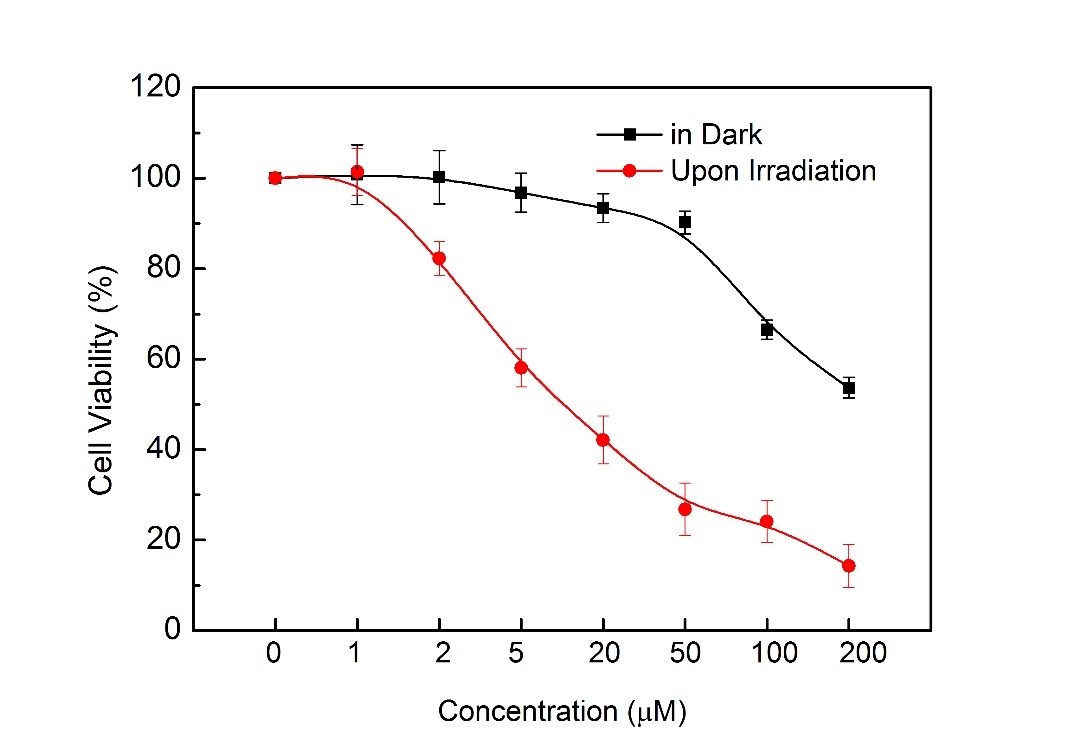


Figure S8 The cytotoxicity of TTRE in 4T1 cells
